# Digital marketing of commercial breastmilk substitutes and baby foods: strategies, and recommendations for its regulation in Mexico

**DOI:** 10.1186/s12992-023-00908-x

**Published:** 2023-02-01

**Authors:** Pedro Javier Mota-Castillo, Mishel Unar-Munguía, Andrea Santos-Guzmán, Marena Ceballos-Rasgado, Lizbeth Tolentino-Mayo, Simón Barquera, Matthias Sachse Aguilera, Fernanda Cobo Armijo, Anabelle Bonvecchio

**Affiliations:** 1 Center for Health and Nutrition Research, National Institute of Public Health, Universidad No. 655 Colonia Santa María Ahuacatitlán, Cerrada Los Pinos y Caminera C.P, 62100 Cuernavaca, Morelos Mexico; 2 Centre for Global Development, University of Central Lancashire, Preston, PR1 2HE UK; 3 United Nations International Children’s Emergency Fund UNICEF, Mexico City, Mexico

**Keywords:** Breastmilk substitutes, Commercial milk formula, commercial determinants of health, Corporate power, Infant formula, Baby food, Digital marketing, Multinational corporations, breastfeeding, international code of Marketing of Breastmilk Substitutes

## Abstract

**Background:**

Parents are exposed to breastmilk substitutes and baby foods marketing on the internet and social media, which hinders adequate breastfeeding and complementary feeding. This study identifies digital marketing strategies for breastmilk substitutes, specifically commercial milk formula and baby foods used by the industry to influence infant and young children’s feeding practices in Mexico and proposes regulatory recommendations that can be useful for similar countries.

**Methods:**

Qualitative study based on the CLICK monitoring framework developed by the World Health Organization, adapted for digital marketing of commercial milk formula and baby foods. Semi-structured interviews (*n* = 53) with key actors were conducted between November 2020 and March 2021, and used grounded theory for the analysis and interpretation with the MAXQDA 20 software.

**Results:**

Commercial milk formula and baby food companies use digital media to contact and persuade parents to use their products by sending electronic newsletters with advertising. Companies hire influencers to market their products because there is no regulation prohibiting the advertisement of breastmilk substitutes on social media, and promote formula among health professionals inviting them to participate in sponsored webinars on infant nutrition, ignoring conflict of interest and the International Code of Marketing of Breastmilk Substitutes. Parents trust formula and baby food advertisements, which use emotional messages and health and nutrition claims to encourage their consumption. Health professionals consider that claims contribute to the indiscriminate use of formula, and some actors propose the use of plain packaging for these products.

**Conclusions:**

Breastmilk substitutes companies promote their products in digital media using unethical strategies that fail to comply with the International Code of Marketing of Breastmilk Substitutes. They generate strong conflicts of interest with health professionals, taking advantage of legal framework gaps and the lack of monitoring and effective sanctions for non-compliers. Updating the legal framework and monitoring compliance, including digital media, is urgently needed to protect children’s right to breastfeeding, healthy nutrition and life, and the rights of women to health and informed decision-making.

**Supplementary Information:**

The online version contains supplementary material available at 10.1186/s12992-023-00908-x.

## Introduction

Breastfeeding and adequate infant and young child feeding practices in the first years of life are essential to prevent malnutrition problems and achieve optimal development [1]. In Mexico, only 28.6% of children under 6 months are exclusively breastfed, 42.9% of children under 12 months consume commercial milk formula, and about 30% of children between 6 to 11 months do not have minimum food diversity [2]. This situation contributes to the triple burden of childhood malnutrition (anemia, undernutrition and obesity) faced by the country [3].

The commercial determinants of health are shaping food systems and have increased milk formula and baby foods sales with an unequal distribution of wealth [4], and in the detriment of breastfeeding practices and maternal and child health [5]. Non-compliance with the International Code of Marketing of Breastmilk Substitutes (the Code) [6] and subsequent resolutions [7] is high in many countries [8]. Breastmilk substitutes (BMS) companies have directed their advertisement efforts towards digital spaces such as social media and internet, using emotional appeals, misleading messages and more persuasive marketing strategies than traditional media [9], that are difficult for parents to identify and for the authorities to monitor and regulate [10]. This has also been pointed-out by the World Health Organization (WHO) multi-country study on BMS marketing [11, 12].

Mexico does not have a law of the Code, but some of its provisions are included in different regulations. The General Health Law on advertising indicates that BMS may not be promoted in health centers [13], and the Regulation for Sanitary Control of Products and Services states that the marketing of infant formulas should promote breastfeeding, indicate correct handling, preparation and care of commercial milks formula and recommend its use only due to breastmilk intolerance, mother’s absence, inability to give milk or any other well-founded health reasons [14]. The official norm NOM-131-SSA1–2012 states that those responsible for the sale or supply of formulas must comply with the Code and subsequent resolutions [15]. Non-compliance with national regulations must be reported to the Federal Commission for the Protection against Sanitary Risks (COFEPRIS by its Spanish acronym). However, there is no monitoring system and sanctions to ensure compliance [8, 16], and many violations have been documented in health services and traditional media [17], as well as on social media and the internet [18–20]. In addition, the BMS industry markets its products through engagement with health professionals [21] generating commercial conflicts of interest that influence the objectivity of their professional performance [22, 23].

Recently, we documented that 93.9% of Mexican parents of young children and with access to internet are exposed to digital marketing of commercial milk formula and baby foods, and those more frequently exposed were 62% less likely to exclusively breastfeed within the first 6 months and more likely to give their children processed foods and sugary drinks [24]. The WHO recommends ending the inappropriate promotion of products that contain added sugars, salt, trans fats, additives, or ingredients not suitable for infants and young children [25], including follow-up and growing-up milks which are in the Code’s scope [26]. These formula milks are ultra-processed products unnecessary for children’s diet [27] which also have a high environmental impact [28]. The WHO recommends implementing a warning labeling system for commercial milk formula and baby foods [29], but in Mexico these products were not included in the front-of-package labeling norm [30], so specific regulations are needed.

This study is independent of the WHO multi-country study [12]. It was funded by UNICEF to identify digital marketing strategies for BMS specifically commercial milk formula and baby foods used by the industry to influence infant and young children’s feeding. This study is innovative since it adds the perspective of different key actors, including parents, health professionals, and representants of civil society organizations, marketing agencies and BMS companies, and proposes regulatory recommendations. We adapted the CLICK methodology developed by the WHO [31], a flexible tool that supports the monitoring of digital marketing of unhealthy products to children. The intention was that the methods used in this research could be used by other countries to regulate the promotion of these products in digital media.

## Methods

This is a qualitative study of interviews conducted with mothers and fathers of children under 2 years of age, health professionals, influencers (people with the ability to influence others through social media) on maternity or parenting issues, and representatives of civil society organizations, marketing agencies, BMS (milk formula) and baby food companies, and the Mexican National Dairy Industry. The participants were selected through intentional sampling [32] to ensure they had the main characteristics of interest and wealth of knowledge about the phenomenon to be studied, in this case regarding digital marketing of BMS and baby foods, as well as breastfeeding and complementary feeding practices.

Parents of children under 2 years of age were invited to participate after answering an online survey about their children’s eating habits and digital marketing of commercial milk formula and baby foods, explained elsewhere [24]. Other key actors were identified based on their influence on breastfeeding and infant feeding issues, their professional or academic networks, in addition to their presence in social media. The selection process is explained in detail in supplementary material. To participate, they were sent an email with the objective of the study, a description of what their participation would entail, an invitation and informed consent letter.

Semi-structured interviews (*n* = 53) were conducted from November 2020 to March 2021. Initially, it was planned to carry out a total of 44 interviews; however, due to the fact that theoretical saturation had not been reached in some topics, that is, when the interviews did not provide new information to the object of study, a greater number of voices and narratives were added to allow a better description and understanding of digital marketing (Supplementary Table 1).

Due to the COVID-19 pandemic, interviews were conducted through the GoToMeeting app and over the phone. The interviews lasted an average of 1 h and were directed mostly by the main author (PM) with a guide reviewed by all the authors of the manuscript, which consisted of five main topics (Table 1). All interviews were audio-recorded and transcribed by the researchers. The interviews were carried out and analyzed in Spanish, and quotes were translated into English by a professional translator for the purpose of this article.

**Table 1:** Main topics included in the interview guide of key actors for the study on digital marketing of commercial breastmilk substitutes and baby foods in Mexico

**Contextual information**	
1. Knowledge about breastfeeding and infant and young child feeding recommendations (given the emerging nature of the topic, we also inquired about breastfeeding and COVID-19).	
2. Knowledge about formulas, beverages and processed and ultra-processed foods for children under two years of age.	
**I. Strategies of digital marketing of formula and baby food on Internet, social media, and websites.**	
3. BMS (infant formula, follow-on formula and growing-up milk) and baby foods marketing on social media, websites or any other digital media.	
4. Differentiation of marketing in digital vs traditional media (i.e., TV, radio, print).	
**II. Conflict of interest.**	
5. Knowledge about conflict of interest and interaction with other actors (this topic was investigated with Influencers, health professionals, representatives of BMS and baby food companies, marketing agencies and civil society organizations).	
**III. Non-compliance with the International Code of Marketing of Breastmilk Substitutes (the Code).**	
6. Knowledge about the Code, perception of compliance/non-compliance and monitoring mechanism.	
**IV. Current regulation of the marketing of BMS and baby foods and recommendations**	
7. Knowledge of existing regulations for marketing BMS and baby foods in digital media (and other media).	
8. Recommendations to regulate digital marketing of BMS and baby foods.	
**V. Health and nutrition claims and front-of-package warning labeling for BMS and baby foods.**	
8. Health and nutrition claims used in BMS and baby foods labeling and marketing.	
9. Front-of-package warning labeling for BMS and baby foods.	
10. Hypothetical scenarios, (i.e., in the case of marketing agencies, examples were included that dealt with how they would design advertising for formula or baby foods or in the case of health professionals, about what would they do in case of a medical consultation to use formulas).	

*BMS* breastmilk substitutes

The analysis of the information was carried out with the MAXQDA 20 software and incorporated agreements between coders to increase its rigor; once at least 80% similarity was obtained between two independent analysts, all interviews were coded. Subsequently, for the analysis, we followed the steps of the grounded theory [33], which consists of building theories, concepts, hypotheses, and propositions, with the data as the starting point. An a priori code book was made, complementing it with the emerging codes by reading the interviews in a continuous and reflective manner. Subsequently, the quotes that best illustrated the findings were retrieved and conceptual maps were constructed to manage the information and better identify the differences and similarities between the perspectives of the interviewed actors.

## Results

### Characteristics of the participants

On average, parents were 31 years old and had two children with a mean age of 1 year and 7 months (Min: 3 months, Max: 2 years). A third of parents were women, who primarily had a medium to high socioeconomic status, as well as university education. They were from different states of Mexico, mainly from the northern region of the country (Table 2). The health professionals, influencers, and representatives of BMS and baby food companies, civil society organizations and marketing agencies are described in Supplementary Table 2.

**Table 2:** Sociodemographic characteristics of parents interviewed for the study on digital marketing of breastmilk substitutes and baby foods in Mexico

Sociodemographic characteristics	Number of participants (***n*** = 27)	%
**Sex**		
Women	20	74.1
Men	7	25.9
**Education**		
Secondary	2	7.4
High school	7	25.9
University	17	62.9
Postgraduate	1	3.7
**Region**		
North	9	36.0
Center	7	28.0
Mexico City	6	24.0
South	3	12.0
Socioeconomic status	11	40.7
A/B		
C	6	22.2
C+	8	29.6
D	1	3.7
D+	1	3.7

Own elaboration with data from the interviews. Socioeconomic status according to the AMAI classification [34]. A/B: Upper Class, C+: Upper Middle Class, C: Middle Class, D+: Lower Middle Class, D: Lower Class

#### Digital marketing strategies for BMS and baby foods

Representatives of BMS and baby food companies and marketing agencies highlighted the use of different digital marketing strategies. They send parents newsletters with advertising through email upon request of information about their children’s age, allowing companies to make product recommendations more accurate. They also mentioned purchasing spaces on podcasts about maternity or nutrition issues, and the “live broadcasts” where a health professional provides seemingly impartial recommendations about infant nutrition to mothers. Companies mentioned collaborating with influencers to promote their products in social media and blogs, as commented by a representative of a BMS and baby food company:

*“ … we do work with a group of influencers, but they cannot promote any product for children under twelve months, [ … ]in fact, as we work with them, they do not mention the product or claims; they simply participate with our experts with a doctor to speak about a topic of general interest for the mother, for example, "stages of development of children over twelve months" and it is nothing more than a topic that goes with branding of the product, but we never send them a sample of the product … .”* (Interview 2, BMS and baby food company)

Commercial milk formula and baby foods marketing is usually present in social media groups such as Facebook and messaging applications like WhatsApp. The mothers stated that nutrition advice in these groups seems useful to them. However, given the diversity of recommendations, they occasionally tend to get confused and doubt the authenticity of the profiles of “mothers” who make insistent product recommendations of a specific brand. This was also mentioned by health professionals. One of the testimonies from a mother serves to illustrate the above:

*“There is a mother who I think that this [formula brand] should pay her some money there, because she always says that “No, that the best is [formula brand] and that [formula brand] has many options, that [formula brand] is this the best for babies”, so she is the only one who is always very attached to formulas and [formula brand], so in fact we already know, we already tell her “the mother [brand of formula] of formula]” [laughs].”* (Interview 16, Mother)

The marketing agencies indicated that most of the advertising strategies are aimed at new fathers and mothers, who have less experience with maternity/paternity issues. They usually look for more information on the internet and are more likely to buy something new, one of the participants commented:

*“ … [about new parents] … they are the ones who are looking the most for information for the first time, that is, and this world of ignorance about being a first-time dad in which it seems that the answer is, obviously, among family members who already have experience or friends or people around, but they also look for many things on the internet or in other places, so they do focus more on them because they are the first ones who would consider trying something new … ”* (Interview 3, Marketing agency)

Another particular target audience for BMS campaigns is working mothers or those who were unable to breastfeed or are undecided about how to feed their babies. Accordingly, companies direct their efforts to advertise the nutrients and components of formulas, as well as the endorsements of health professionals, to convince them that commercial milk formula is safe, and sometimes even better than breastmilk.

*“ … , there were mothers who for different reasons could not breastfeed their babies or did not want to do so and needed to feel safe and calm that not doing so was not causing harm to the babies and so this is where it became relevant to talk about the nutrients and talk to them about the components of the formulas, and talk to them about all these things that helped them calm down a little bit, "it's okay, and that's fine, if you can't feed your baby, that's fine, you can give him formula and the pediatricians they endorse you and absolutely nothing happens, you are not a bad mother for doing it … ”* (Interview 5, Marketing agency)

The marketing agencies pointed out that, for the design of advertising messages, mothers are usually divided into two types. The first alludes to the most emotional issues of the mother-child relationship, so the advertising refers to maternal care and love, while the second is much more informative and the message is about the nutritional content of the products, as one publicist described it:

*"…yes, it's very empathetic, so to speak, they try to be empathic, but on the other hand, I think that this type of product also requires a lot of information, that is, much more specific, that is, yes, people who have the opportunity to know what they are giving their babies, so either they have this area that is much more sentimental and emotional or this area that is much more informative, but I think it is divided into those two big ones … ”* (Interview 3, Marketing agency)

This type of marketing makes parents trust that the advertised product is good for their child, and they believe that it should not contain anything that could cause harm. They also point out that none of the companies allude to benefits that the product does not have. One of the parents expressed:

*"...the truth is that the information that I have found regarding my child's situation is very abundant, with all the scientific limitations that I may have, fully trusting that what they are offering us is really what it is, the decisions have been made … I tell you; I completely trust what they offer us”* (Interview 4, Father)

### Conflict of interest

**Fig. 1:**
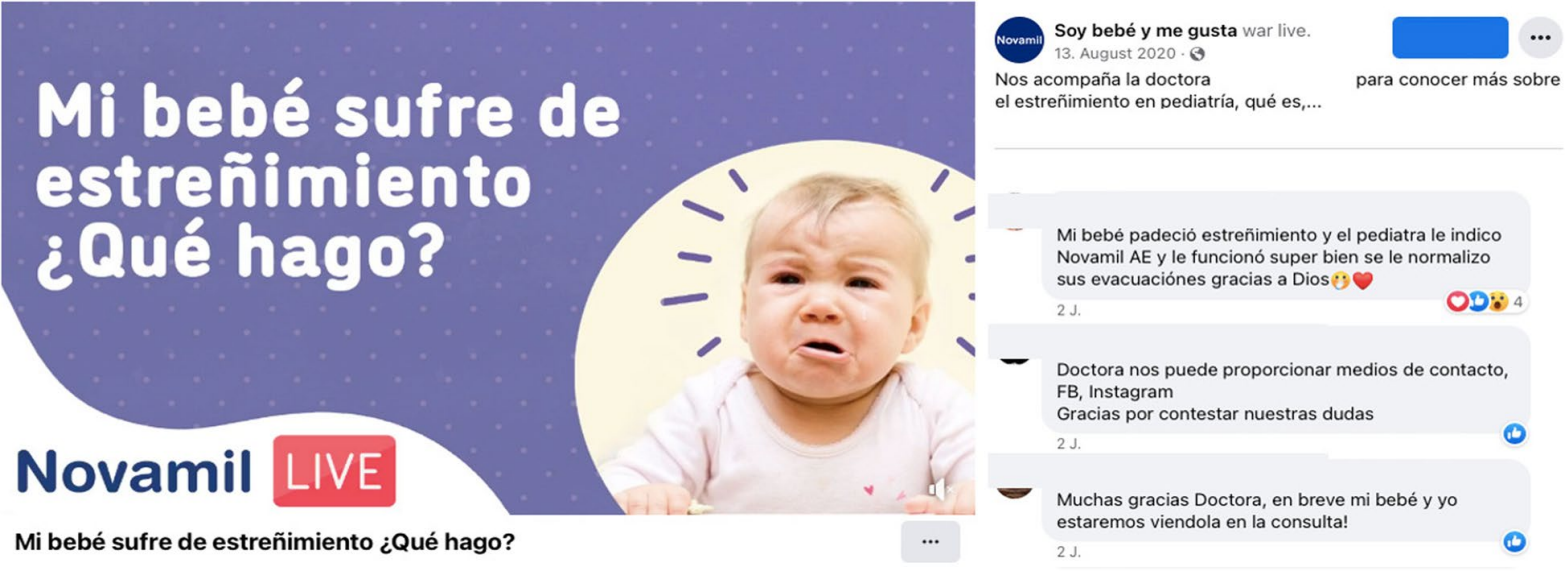
Endorsement of a product and participation of health professionals in webinars sponsored by breastmilk substitutes companies in Mexico. Official webpages and social media of breastmilk substitutes companies in Mexico

Influencers, and health professionals were unaware of the potential conflict of interest of engaging with the industry of BMS. Industry representatives reported that the events they organize, such as congresses, live broadcasts, and educational webinars incorporate impartial recommendations on breastfeeding and complementary feeding, even when the brand is paying for the space (Fig. 1). The health professionals considered that there is no conflict of interest in collaborating with the BMS companies, since they do not give a direct suggestion or recommendation of their product that would undermine their credibility with their patients, despite appearing in video segments sponsored by the brand. One of the participants described:

*“I work as a science speaker, that is, sometimes I am invited to different forums, some subspecialist pediatric conferences, and I talk a lot about complementary feeding, but from the science side, never, never, never promoting the brand or mentioning any type of product advantage, etc.”* (Interview 2, Health professional)

Some representatives of civil society organizations mentioned that, in previous years, due to the lack of government support and resources, they had to accept financing from BMS and baby food companies, in exchange of training, as described by one of the participants:

*“Once, [brand] offered me to train people, there was a time when we were training and supported by [name a different brand], although we always restricted everything a lot, they supported us, but at this time we no longer have support from anyone. Sometimes even a person [NAME] who is [position] of the network that monitors the International Code in Latin America complained to me and said "hey, why does [brand] support you?" and I told him because there is no one else to support us, we cannot do things alone”* (Interview 1, Civil Society Organization)

### Non-compliance with the international code of Marketing of Breastmilk Substitutes

BMS and baby food companies mentioned that they only advertise products that they consider to be outside the scope of the Code provisions and national regulations; for example, porridge and growing-up milk for children 1 year old or older, as expressed by one of the participants:

*“ … [about the products they advertise] the rest of the portfolio that is not within the World Health Code, that is not in that scope would be, for example, our porridge as [Brand], everything that is the [Brand] line or the growing-up formulas that are all those products that you can give the child from the year onwards, there we do advertise … ”* (Interview 1, BMS and baby food company)

The health professionals pointed out that they have reported some of the non-compliances to the Code directly with the BMS companies, obtaining a vague response, as reported by one participant:

*“ … I once wrote to [Brand], because I was ashamed to see one of their ads and they told me like -well yes, what you think is valid, but that's the way it is-, then really in Mexico there is no respect for the Code, … we cannot expect good things and if the companies do not pay attention to it, they do what they want.”* (Interview 1, Health professional)

The representatives of the civil society organizations mentioned that they usually monitor compliance with the Code, including in digital media, and document their non-compliance by reporting it to the International Baby Food Action Network (IBFAN), and health professionals also evidenced the lack of knowledge of a regulatory body (COFEPRIS) that is in charge of monitoring the advertising of BMS and baby foods to report breaches of the Code contained in national legislation. One of the participants narrated:

*“Yes, also [digital marketing is monitored] I tell you, in this YouTube video, I took a screenshot and we sent it, or when I get advertising from a congress or from a doctor who is part of a formula company, also, that is everything, everything, that is what you see in the supermarket and also what you see on social networks, all this is sent to IBFAN.”* (Interview 4, Civil Society Organization)

Health professionals commented that there is a lack of knowledge about the Code, and in a testimony, it was perceived that from early stages of their training as physicians, the BMS industry sponsors congresses and trips, thus creating brand engagement, and seeking the gratitude of the health personnel by prescribing their products.

*“I think the industry’s impact is more with physicians. For example, I can tell you that, in my residence in [hospital’s name], they [the industry] took us to Acapulco on a trip, and well with all expenses paid for residents to attend events, and clearly the fact that the industry pays for that is an infatuation for those who are in diapers, and you come out with that … and no one is aware of the Code, so much, that when you tell them about the Code, people think you are crazy.”* (Interview 5, Health professional)

### Current regulation of the marketing of BMS and baby food and recommendations

Health professionals, marketing agencies, influencers and companies identify the regulation of marketing as a pending issue. The existing regulations are seen as obsolete and many times they are not respected by BMS and baby food companies when promoting their products. The Regulation for Sanitary Control of Products and Services of COFEPRIS has not been modified for the last 20 years. Companies and marketing agencies know that less attention is paid to digital marketing, since COFEPRIS cannot monitor all the material that is published every day on the different social media platforms, as one publicist mentioned:

*“COFEPRIS has a regulation that has been in effect for twenty years, and well, every time you enter a category you have to look for that file and understand what the regulations and restrictions are, normally, especially the categories that are known to be punished the most, for example formulas, for example pharma [pharmaceutical], for example alcohol, they themselves have a small regulatory department as a client that more or less has an understanding and has a good reading of this type of document and then from the moment you receive the brand they themselves tell you what they can say and that they cannot say and you can find just examples or ways to turn them around on how you could say something without saying no and that is where unbranded content and content with influencers are born, right? because there are also legal loopholes in the things you can or cannot say … ”* (Interview 5, Marketing agency)

The influencers also commented that there is no legal instrument for regulating digital marketing in Mexico, since anyone can promote anything on social media, as one of them mentioned:

*“No, no, there is nothing, [laughs], no, there is nothing to regulate, that is, even anyone can talk about it [referring to infant feeding] and if you are like an actress or something like that, that in fact they have already been doing it because they can pay them a good deal, make videos, events, everything, without limit.”* (Interview 1, Influencer)

### Health and nutrition claims and front-of-package warning labeling

Marketing agencies and companies were asked about the possibility of a front-of-pack warning labeling for commercial milk formula and baby foods, to which both groups expressed concern and argued against it. These actors considered that food decisions can improve with more nutrition education, not with the implementation of a policy such as labeling.

*“ … I think it is a matter of education. I think it's more a matter of education beyond whether or not labeling, right? The issue of "Excess of" doesn't seem to me to make any sense, excess of, compared to what, but well, that's my perception...”* (Interview 1, BMS and baby food company)

Parents approved the possible implementation of a warning labeling for commercial milk formula and baby foods. They suggested that it would be useful for them to quickly identify the components of the product and choose the foods that they will offer their children, as one of the mothers expressed it:

*“[on the usefulness of labeling in infant food products] Well, in really realizing what the formula is made of, what it has, -excess sugars-, -excess sodium-, what it contains, I imagine that with the labels there, you really look at what there is, what it has, and well, one already makes a little more conscience in deciding it.”* (Interview 8, Mother)

On the other hand, informants identified that the lack of a warning labeling for food and beverages marketed as suitable for children under 3 years influences the decisions of parents when choosing the foods to feed their children. This is important since parents think that baby foods are also included in the scope of the current warning labeling, confusing them at the time of purchase:

*“ … The pouches, in fact, we continue to give them (to our child) because you see that in the Government’s labeling it says “Excess of sugars” or something like that, and in the [brand] pouches there is no warning … I always look at the packages and, for example, if it does not have the warning seal, we want to assume and believe that they are natural foods, and that they feed (our child) properly, so that’s why we continue to buy that kind of foods … If it says natural fruit and without preservatives, then that’s why we decide to keep buying it”.* (Interview 7, Father)

**Fig. 2:**
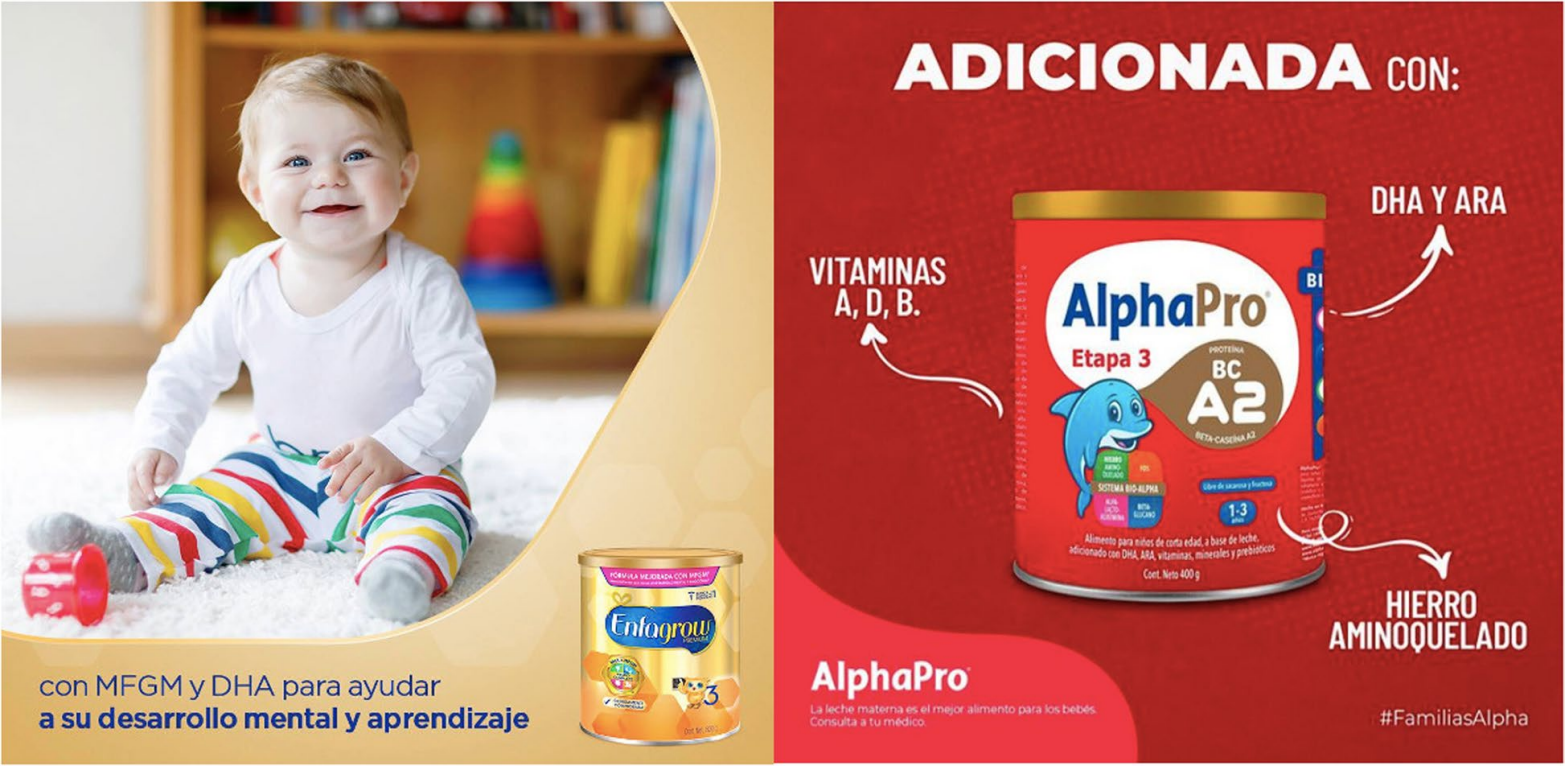
Health and nutrition claims on a commercial milk formula in Mexico. Official webpages and social media of breastmilk substitutes companies in Mexico

Health professionals consider that health and nutrition claims used in formula contribute to its indiscriminate use (Fig. 2). The representatives of the civil society organizations agreed with the health professionals, and spoke in favor of a clear labeling that is aligned with the Code. From their perspective, a plain packaging, without images or health and nutrition claims, would help to reduce the indiscriminate use of BMS.

*“ … I think that this type of warning could be totally considered and if not … use strategies that are more oriented towards plain packaging, that is, that they cannot use this type of persuasive elements that we have been talking about, that they cannot say -for smarter children-, -for healthier children-, -for children without colic-, all these allegations”* (Interview 2, Civil Society Organization)

## Discussion

This study aimed to identify digital marketing strategies of BMS, specifically commercial milk formula and baby foods in Mexico, used by the industry to influence parents and caregivers about their infant and young children’s feeding habits and propose recommendations for its regulation.

The main digital strategies include sending electronic newsletters with advertisement of commercial milk formula and baby foods to parents via email, through which companies collect personal information, promoting online parent clubs, collaborating or hiring influencers in social media and engaging with health professionals to promote their products, which were also found by the WHO multi-country study [11].

Companies use unethical promotional strategies in digital media that can be segmented according to the targeted population or “personas”, which are fictional profiles that represent groups of similar people in a target audience, to reach people on a more personal level, through messages, offers and the right products at the right time. Using social media data to target mothers more precisely by milk formula companies has been reported by Hastings et al. [35], and is part of the ‘surveillance capitalism’ where raw personal data is monetized for hidden commercial purposes and used for conditioning people’s behavior [36], increasing the corporate power of BMS industry.

Also, we found that commercial milk formula and baby food companies sponsor conferences, webinars, and other child health and nutrition events to position as an infant feeding advisor and educator, and engage with health professionals to conduct online events where they recommend their products, leading caregivers to believe that BMS have scientific support, and that it provides the same benefits as breastmilk. During 2021, the main formula company in the country launched an educational program on parenting and nutrition in the first 1000 days of life [37], interfering with adequate infant feeding practices. Medical marketing has been documented in Philippines were BMS industry have sponsored pediatric, midwives and nutritionist associations [38], and also by the WHO multi-country study [12]. This collaboration cannot be conceptualized as impartial or objective, as companies would have us believe. The events are plagued by the brand’s logo and slogan, which represents an “endorsement” by association, one of the main strategies of the marketing industry [35]. From the testimonies we collected, conflict of interest generated by the industry is often unrecognized, or it does not seem to be perceived as a problem or as a lack of compliance with the Code. However, promoting and endorsing the consumption of BMS and baby foods interferes with respect to a primary public health interest such as breastfeeding and adequate complementary feeding practices, violating the right of every child to reach their full potential of healthy nutrition during their first years of life and women’s right to informed decision-making [22, 23].

A key finding is the diversity of positions on the regulation of digital marketing of milk formula and baby foods, since civil society organizations, health professionals and influencers point out the lack of regulation in digital media, while BMS companies mention that regulations in Mexico are sufficient. Although companies say they agree with the Code, actually they only respect what is effectively regulated and sanctioned at the local level, so accepting the Code at the international level does not commit them to comply with it. However, national regulations have shortcomings in terms of the promotion and commercialization of milk formula and baby foods and loopholes in the legislation allow companies to market their products even though they are violating the Code.

These results are similar to those found in other countries, where violations of international and national legislation regarding digital marketing of BMS are frequent and difficult to regulate by the authorities. Failure to comply with regulations are observed mainly through online baby clubs, articles with educational information and direct messages through WhatsApp and content on social media [11, 39, 40].

Company’s representants declared that they promote only baby foods and growing-up milk for children from 1 year of age, which they perceive to be outside the scope of the Code. Growing-up milks are increasingly being marketed and recommended by health professionals in Mexico, making women believe their children need these products [41]. Widening the range of milk formulas for older children have been documented as part of the strategies used by industry to face regulations they perceived as applying only to infant formula and expand their business [5, 38]. It is necessary to use information such as that presented in this study and in the WHO multi-country study to show violations of the Code, since other indexes such as the Access to Nutrition Initiative rate many companies well despite their non-compliance every year [21].

Health professionals and civil society organizations identified health and nutrition claims as a factor that contributes to the abuse of formulas for misdiagnosed conditions, so they proposed it be regulated and suggested a plain packaging. A study in the United States found that health and nutrition claims increase the chances that a child will be offered formula, since most caregivers believe in claims found in advertising and over one-half of them agreed that formula can be better than breastmilk [42]. This is consistent with the testimonials of parents in our study, who trust advertising and expect the nutrition claims they read on formula cans or see in a social media post about the product to be true. The WHO recommends plain packaging for commercial milk formula, prohibiting the use of images and text that suggest formulas are equivalent or superior to breastmilk, health or nutrition claims, as well as endorsement by health professionals or organizations [12], also recommended in Hong Kong [43] and suggested by some actors in South Africa [44].

Opinions were divided on front-of-package warning labeling for commercial milk formulas and baby foods, which are not included in the current labeling regulation in Mexico [30]. While the companies and marketing agencies were against it, parents positively valued the possible implementation of this public policy, since it would be useful to them when choosing the foods that they will offer their children. Formulas contain excess sugars [45], and many baby foods contain added sugars and salt, as well as unhealthy fats [46] that increase the long-term risk of poor metabolic health and chronic diseases [47], so it is crucial to define a nutrient profile for a warning labeling and end the inappropriate promotion of these products [25].

### Recommendations for strengthening Mexican regulations to end the inappropriate promotion of commercial BMS and baby foods

Recommended regulations are summarized in Table 3 and were developed by the research team after interpreting the results, and considering the Code’s provisions, subsequent resolutions and recommendations by the WHO.

**Table 3:** Digital marketing strategies of commercial breastmilk substitutes and baby foods and recommendations for strengthening regulations in Mexico

Digital marketing strategies	Description	Current Mexican Regulation	Recommendations and regulation proposal
Peer recommendation in digital media	Anyone who posts or comments in favor of commercial milk formula and baby foods on social media (i.e., Facebook, Instagram) hired by companies.	Not contemplated	The General Health Law on advertising should: Include within the definition of advertising the positioning of products and the use of influencers in digital media and social media. Make the declaration of advertising content in digital media mandatory when receiving any payment or benefit for recommending a product. Train influencers about the Code and Mexican regulations.
Influencers	Influencers on social media post videos, photos and any other material on their accounts where they directly or indirectly recommend commercial milk formula or baby food products so that parents or caregivers of infants and young children, who seek to imitate their lifestyle, consume these products.		
Podcast	Marketing and promotion for commercial milk formula and baby foods on music streaming platforms.		
Mailing /newsletters	Subscription to electronic newsletters with information on food and nutrition, where personal data such as the baby’s date of birth is shared. In this way, companies promote their products according to the age of the baby.	The General Health Law on advertising states that BMS may not be promoted in health centers but does not contemplate the promotion in traditional or digital media.	The General Health Law on advertising and the Regulation for Sanitary Control of Products and Services should: Prohibit the marketing of any BMS, and those baby foods with added sugars, salt, trans fats, excess fats, additives, or any ingredient not suitable for infants and young children in any media, including digital media. Prohibit contact of commercial milk formula and baby food companies with pregnant women, mothers, fathers, caregivers of infants and young children, and health professionals, including digital media. Prohibit BMS and baby food companies from creating, sponsoring, and/or disseminating educational material related to breastfeeding, food, nutrition, and child health in any media.
Online parent clubs promoted by formula and baby food companies	Aimed at new mothers and fathers who sign up for these clubs to receive child nutrition orientation and by doing so expose themselves to the use of their personal data for targeted marketing of commercial milk formula and baby foods.		
Companies pay for displays during navigation on Internet, or spaces in specialized magazines/blogs	When searching for an infant feeding concern on the internet, parents often come across a website for a formula and baby food brand, as these companies pay for their products to appear in searches and on electronic magazines/blogs.		
Sponsorship by companies of educational material, lives, webinars, congresses, and events related to health and nutrition topics	Companies engage with health professionals to give webinars, live broadcasts, videos, and virtual educational material, among others, or events that are sponsored by companies where commercial milk formula and baby food products are promoted.		
Images that idealize the use of formulas, health and nutrition declarations, and endorsement by health professionals of commercial milk formula and baby foods.	Nutritional claims in commercial milk formula that refer to its nutritional content, suggesting the product is similar or better than breast milk. Health claims that indicate that the product is recommended for the relief of a specific disease or symptom (i.e., gastrointestinal symptoms and other common ailments in babies) Images with graphics, pets, landscapes and other figures idealize these products’ consumption.	The Regulation for Sanitary Control of Products and Services indicates that marketing of infant formulas should promote breastfeeding, indicate correct handling, preparation and care of commercial formula milk, and recommend its use only due to breastmilk intolerance, mother’s absence, inability to give milk or any other well-founded health reasons. The official norm NOM-131-SSA1–2012 indicates that those responsible for the sale or supply of formulas must comply with the Code and subsequent resolutions and that formulas must not display images or text that suggest they are identical and superior to breastmilk, nor display nutritional or health claims.	The Regulation for Sanitary Control of Products and Services, and the NOM-131-SSA1–2012 should indicate: Plain packaging for BMS and front-of-package warning labeling about the content of sugars, sodium, and fats in formulas and baby food. No product should include any imagery that could undermine or discourage breastfeeding, make a comparison to breast milk, or suggest that the product is nearly equivalent or superior to breast milk. Do not allow claims of nutritional and health properties and the endorsement of any health professional or organization to commercial milk formula and baby foods. Define a nutrition profile for baby foods to
Implement formal mechanisms for reporting violations of the Code and Mexican regulations, monitor marketing, including digital media, and increase sanctions against the industry when they fail to comply.			

Source: Own elaboration with testimonies and proposals from key actors and recommendations by the World Health Organization

*BMS* breastmilk substitutes

The promotion and commercialization of BMS and baby foods must be regulated, monitored and sanctioned in case of non-compliance, with the aim of protecting the health of children [29]. These penalties must be significant compared to the sales of their products. For this, updated legal instruments are required. They should include the different strategies used in digital media and be aligned with the Code and recommendations from international organizations such as WHO and UNICEF. In this sense, sponsorship, publicity, and advertising promotion of BMS should be prohibited in all media, as well as images, nutrition and health claims, and the endorsement or organizations and health professionals.

There should be no contact, including digital media, between BMS and baby food companies and pregnant women, mothers, fathers and caregivers of infant and young children. Also, companies should not be allowed to sponsor health professionals or events related to nutrition and infant feeding, nor develop and disseminate educational material related to breastfeeding, nutrition and infant health in any media.

It is important to implement codes of ethics, such as the NutriCOI [48] developed by many actors in Mexico and international organizations which seeks to encourage professionals and other sectors related to the problem of malnutrition to conduct themselves in an ethical, transparent and professional way in the face of a potential conflict of interest with the industry and thereby ensure the protection of children’s health and nutrition.

It is essential to limit the interference of the industry in areas such as the academic training of health professionals, as well as in the implementation of educational programs for parents and caregivers of infant and young children which interferes with the decision to breastfeed and with adequate infant feeding practices.

### Strengths and limitations

We recognize that a limitation of this study is that some actors were not considered, so it is desirable to complement this perspective with the voice of other actors involved, such as managers, health students, directors of regulatory agencies and policymakers. However, one of the strengths is that we included industry representatives, marketing agencies and influencers who are key actors in the digital marketing ecosystem, often left out in other studies due to the challenge of identifying and contacting them, and agreeing to participate.

We opted to use the semi-structured interview to give priority to the speeches of the participants and focus on main aspects of digital marketing of BMS and baby foods, at the expense of other elements that could provide understanding of marketing of these products, for example in other media. Since digital marketing is constantly changing and new strategies are rapidly implemented, these may not have been explored in this study in addition to those documented in this article. Although face-to-face interviews would have provided greater inputs to contrast qualitative information, perhaps many key actors would not have participated, hence virtual interviews helped us identify the important practices used in digital marketing.

On the other hand, one of the main strengths of the study is being a pioneer in adapting the first phase of the CLICK monitoring framework to assess parents and other key actors experience and awareness of digital marketing strategies of BMS and baby foods. In addition, we incorporated different voices involved in digital marketing including key actors who market, advertise and prescribe the product to the final consumer, thus providing evidence for public policy and the regulatory instruments that urgently need renewal.

The Mexican case can serve as an example to other countries in the Latin American region to evidence the strategies that companies use in digital media, probable very similar, to persuade mothers and fathers to buy their products and the actions that need to be made to regulate the inappropriate promotion of BMS and baby foods.

## Conclusions

This qualitative study with parents, health professionals, influencers, marketing agencies, civil society organizations, and industry provided insights into the current situation in Mexico where parents are exposed to digital marketing of BMS, especially commercial milk formula and baby foods. Legal instruments have loopholes and are outdated, and are not fully aligned with the International Code of Marketing of Breastmilk Substitutes, which has led companies to continue to breach it using unethical and misleading strategies. Digital marketing of BMS is part of the commercial determinants of health that are shaping the first food system and represent a violation of the right of children to reach their full potential through healthy nutrition during their first years of life and women’s right to make an informed decision about their infant’s feeding. Strengthening legal instruments should be a priority on the country’s political agenda in order to: prohibit and regulate the sponsorship, advertising, and promotion of BMS, including digital media such as the Internet, websites and social media; ban contact of companies with pregnant women, mothers, fathers and caregivers of young children; prohibit the use of nutritional or health claims and the endorsement by organizations or health professional of BMS and baby foods; avoid free delivery of these products, including in emergency situations; and sanction those who fail to comply.

## Supplementary Information

**Additional file 1: Supplementary Table 1.** Invitations to key actors, number of interviews planned and carried out for the study on digital marketing of commercial breastmilk substitutes and baby foods in Mexico. **Supplementary Table 2.** Professional training of participants by type of key actor interviewed for the study on digital marketing of commercial breastmilk substitutes and baby foods in Mexico.

## Data Availability

The information generated and analysed during the current study are not publicly available because of the need to protect participant privacy.

## Abbreviations

BMS: Breastmilk substitutes
Code: International Code of Marketing of Breastmilk Substitutes
WHO: World Health Organization
IBFAN: International Baby Food Action Network
COFEPRIS: Federal Commission for the Protection against Sanitary Risks
