# Supplementary material for: Digital marketing of commercial breastmilk substitutes and baby foods: strategies, and recommendations for its regulation in Mexico
Source: Global Health. 2023 Feb 1;19:8. doi: 10.1186/s12992-023-00908-x (PMC9890409; doi:10.1186/s12992-023-00908-x)
Supplement: Supplementary file 1 — Additional file 1: Supplementary Table 1. Invitations to key actors, number of interviews planned and carried out for the study on digital marketing of commercial breastmilk substitutes and baby foods in Mexico. Supplementary Table 2. Professional training of participants by type of key actor interviewed for the study on digital marketing of commercial breastmilk substitutes and baby foods in Mexico. [file 12992_2023_908_MOESM1_ESM.docx]

**Supplementary material**

**Title:** Digital marketing of commercial breastmilk substitutes and baby foods: strategies, and recommendations for its regulation in Mexico.

**Participant selection process**

We selected marketing agencies from the ranking of the best agencies published in 2019 by the magazine Merca 2.0 (Noticias, 2020), and also those that reported having collaborated in the campaigns of formula and baby foods brands. Two rounds of invitations were sent, since we did not obtain responses from some agencies. We also used snowball sampling, asking those agencies that agreed to participate about other agencies that collaborated in some food advertising campaigns.

In the case of influencers, a mapping of the principal maternity blogs in Mexico was carried out using Starngage (Starngage, 2020.), an influencer marketing platform. The influencers with the largest number of followers on Instagram were identified. After this, we checked that they shared content about infant feeding and that they had posted advertisements with formula and baby foods brands in their blogs or social media (Facebook, Twitter, TikTok, YouTube). On some occasions it was not possible to access the contact information of the influencers in order to invite them to participate in the study, so another influencer with contact information available was invited. In addition to what has already been described, the influencer moms who appeared in the videos of formula and baby foods brands were invited to participate. For health personnel, the Heepsy virtual tool (Influencers, 2020) was used at first with the term pediatrics, after which a search was carried out on Facebook, Instagram and YouTube and those health professionals whose profiles had the highest number of followers were invited to participate. In a complementary manner, health personnel who collaborated in video capsules of some formula and baby foods brands or who were within the portfolio of pediatricians in the brands' blogs were identified and invited to participate.

To identify formula and baby food companies, the three brands and companies with the highest consumption and sales in the country were identified using data from the National health and Nutrition Survey 2018-19 (ENSANUT by its Spanish acronym) (Shamah-Levy et al., 2020) and Euromonitor (Euromonitor, 2020). Then, mapping was carried out through LinkedIn to identify people who worked in the marketing or advertising areas of these companies. On the other hand, an article which mapped and described the influence of civil society organizations on breastfeeding policies in Mexico was used to identify them (Buccini et al., 2020) and preliminary list was made. Those organizations with the largest number of followers on the main social networks (Facebook, Instagram, Twitter and YouTube) were selected.

Supplementary table 1. Invitations to key actors, number of interviews planned and carried out for the study on digital marketing of commercial breastmilk substitutes and baby foods in Mexico.

| **Key actor** | Invitations | Planned interviews | Interviews carried out |
| --- | --- | --- | --- |
| Marketing agencies | 10 | 3 | 5 |
| Formula and baby food companies^a^ | 16 | 3 | 4 |
| Influencers on social media | 9 | 3 | 4 |
| Civil society organizations | 13 | 5 | 7 |
| Health professionals | 15 | 4 | 6 |
| Fathers with children under two years old | 18 | 6 | 7 |
| Mothers with children under two years old | 38 | 18 | 20 |
| Total | 119 | 44 | 53 |

1. Includes a body that represents milk producers in the country

Supplementary Table 2. Professional training of participants by type of key actor interviewed for the study on digital marketing of commercial breastmilk substitutes and baby foods in Mexico.

| **Key actor** | **Interviews carried out** | | **Professional training of participants** |
| --- | --- | --- | --- |
| Marketing agencies^1^ | 5 | 2 publicists, 2 visual artists, 1 communication specialist, 1 graphic designer | |
| Formula and baby food companies^2^ | 4 | 1 Neurobiologist, 1 Publicist, 1 Hotel Business Manager, 1 Agricultural Engineer | |
| Influencers in social media | 4 | 2 educators, 1 communication expert, 1 Master in digital media | |
| Civil Society Organization | 7 | 2 Nutritionist, 1 Breastfeeding Specialist, 1 International Relations, 2 Pediatrician, 1 Ethnologist, | |
| Health professionals | 6 | 2 Gastropediatricians, 1 IBCLC*, 1 Nutritionist, 2 Pediatrician | |
| **Total** | 26 |  | |

Elaborated with data from the recruitment of participants. 1. one of the interviews carried out with two people at the same time, for this reason, even if there are 5 interviews, 6 profiles are shown. 2. Includes a body that represents milk producers in the country. *IBCLC: International Board-Certified Lactation Consultant

**References**

Buccini G, Harding KL, Eguiluz IF, Safon CB, Hromi-Fielder A, Cosío TG De, et al. An analysis of stakeholder networks to support the breastfeeding scale-up environment in Mexico. J Nutr Sci 2020:1–10. https://doi.org/10.1017/jns.2020.4.

Euromonitor. Euromonitor International | Strategic Market Research, Data & Analysis 2020.

Influencers H. Heepsy 2020. https://www.heepsy.com/?utm_source=google&utm_medium=cpc&utm_campaign=search_en_brand&gclid=Cj0KCQjwma6TBhDIARIsAOKuANxlCDbB5ALKpEugo5h-wCYbqpDT7XzXdYhm8FkklSWprXbtmABxkNwaAiJqEALw_wcB.

Noticias MPM. Merca 2.0 2020. https://www.merca20.com/.

Shamah-Levy T, Vielma-Orozco E, Heredia-Hernández O, Romero-Martínez M, Mojica-Cuevas J, Cuevas-Nasu L, et al. Encuesta Nacional de Salud y Nutricion 2018-19 Resultados nacionales. vol. 53. 2020.

Starngage. 2020. https://starngage.com/app/global.
